# Identifying Perceived Barriers to Human Papillomavirus Vaccination as a Preventative Strategy for Cervical Cancer in Nigeria

**DOI:** 10.5334/aogh.2890

**Published:** 2020-09-17

**Authors:** Nicole Yvonne Nguyen, Emeka Okeke, Andrew Anglemyer, Tina Brock

**Affiliations:** 1 Department of Clinical Pharmacy, University of California, San Francisco, California, US; 2 RISE Clinic Nigeria, Agulu-Nnobi Road, Adazi-Ani, Anambra, NG

## Abstract

**Background:**

Cervical cancer deaths are disproportionately higher in developing countries depicting one of the most profound health disparities existing today and is ranked as the second most frequent cancer among women in Nigeria. The Human Papillomavirus (HPV) vaccine as a primary prevention strategy is not widely used in Nigeria. This study investigated perceived barriers to HPV vaccination in a Nigerian community, targeting health workers’ perceptions.

**Methods:**

This descriptive study captured responses from a cross-sectional, convenience sample of adult health workers within Anambra State, Nigeria. An anonymous 42-item survey with multiple validated scales was developed based on the Theory of Planned Behavior model and previous studies. The self-administered survey was distributed by research assistants at study sites within Anambra State which were identified through local constituents by the regional zones Adazi-Ani, Onitsha, and Awka. Data analyses were performed using Microsoft Excel for descriptive statistics and R software for the logistic regression, with a statistical significance level of 5%. Subgroup analysis was performed for the baseline knowledge questionnaire to determine if there were any differences in correct responses based on demographics such as: Institution type, profession, age, sex, religion and parental status.

**Results:**

Responses were collected from 137 Nigerian health workers; 44% nurses, 14% physicians, 6% pharmacists and 31% other health workers. The majority of respondents were female (69%), between 18 and 39 years of age (78%), from urban settings (82%), and identified as having Christian religious beliefs (97%). The most significant barriers identified were lack of awareness (39%), vaccine availability (39%), and cost (13%). When asked baseline knowledge questions regarding HPV, females were more likely to answer incorrectly as compared to males. Significant differences were found for statements: (1) HPV is sexually transmitted (p = 0.008) and (2) HPV is an infection that only affects women (p = 0.004).

**Conclusions:**

Perceived barriers to HPV vaccination identified by Nigerian health workers include lack of awareness, vaccine availability/accessibility, cost, and concerns about acceptability. Ongoing efforts to subsidize vaccine costs, campaigns to increase awareness of HPV vaccine, and interventions to improve attainability could advance administration rates in Nigeria, and ultimately improve death rates due to cervical cancer in this population.

## Background

Death from cervical cancer is a profound health disparity in developing countries compared to developed countries which must be addressed on a wide scale. It is estimated that approximately 85% of all cervical cancer deaths occur in developing countries, where women often lack access to cervical cancer screening, HPV vaccination, and treatment [1,2].

Human papillomavirus (HPV) infection is a well-established cause of cervical cancer. Infection can be identified through screening and prevented through vaccination. Cervical cancer is the second most frequent cancer among women in Nigeria [3]. There are an estimated 53 million women, ages 15 years and older, at risk for cervical cancer in Nigeria [4]. The World Health Organization’s Information Centre on Human Papillomavirus (HPV) and Cervical Cancer estimates that every year 14,550 women in Nigeria are diagnosed with cervical cancer and that 9,649 die annually from the disease [3]. In 2011, Nigeria implemented a national strategy to reduce the mortality associated with cervical cancer, demonstrating that this has been identified locally as a key health concern, but plans for largescale vaccination have yet to be established nationally [5].

Primary prevention of cervical cancer is achievable through widespread education, annual pap smears, and vaccination. Human papillomavirus (HPV) serotypes 16 and 18 cause 70% of cervical cancer cases [6]. The bivalent HPV (types 16, 18) vaccine is currently licensed for use in Nigeria and is an effective strategy to preventing HPV infection [7]. In 2010, efforts were reportedly being made to increase accessibility and affordability of the vaccine through negotiations with drug manufacturers and the Global Alliance for Vaccines and Immunization (GAVI), a public-private partnership dedicated to ensuring worldwide access to vaccines [7]. However, the HPV vaccine has yet to see widespread use as a routine vaccination in this population. The reasons for this are unclear. Cultural differences could potentially complicate widespread vaccine use in Nigeria due to a historical mistrust of western medicine which has contributed to the failure of some polio vaccine initiatives in northern Nigeria [8]. An HPV and Related Diseases Report was published by the National HPV Immunization program in Nigeria in 2018, unfortunately there was no available data regarding HPV vaccination coverage rates nor was there a national strategy in place for targeted populations for vaccination [9]. While recent reports aim to summarize the burden of disease in Nigeria for stakeholders, there is a gap in knowledge regarding the barriers to uptake of the known prevention strategy of vaccination.

The purpose of this study was to investigate the perceived barriers to HPV vaccination, targeting health workers’ perceptions within Anambra State, Nigeria. Investigating what barriers exist to vaccination can help stakeholders create targeted strategies and recommendations to further promote widespread use of this life-saving vaccination.

## Methods

### Study Design

This descriptive study utilized a cross-sectional, convenience sample. An initial survey questionnaire was developed based on an extensive literature review which identified multiple validated scales. Questions were developed based on the Theory of Planned Behavior model and previous studies [9,10,11,12]. Following a pilot study period which included 22 respondents within the target population sample, a finalized 42-item anonymous survey questionnaire was developed. This study was classified as exempt from full review by the Institutional Review Board [13].

### Data Collection

The finalized survey was distributed by research assistants at the study sites and participants independently completed paper versions of the questionnaire (self-administered survey). The survey combined multiple-choice and free response questions that collected data including demographics, clinical experience and practices, perceptions, and baseline knowledge (see Appendix 1 for survey questions). Inclusion criteria were ≥ 18 years of age, health workers working within Anambra State located in the south-east region of Nigeria.

### Study Area

Anambra state, the second most densely populated state in Nigeria, is reported to have the highest literacy rate in the country and ranks high in terms of education. This state was chosen as a study site due to local ties and established educational relationships among participating programs.

Anambra State is predominately Christian as are other States in Southern Nigeria. Minimum wage in Anambra State is N18,000 (Nigeria Naira), or $50 monthly (US), as of 2018 [14]. These numbers apply mainly to civil servants employed in the public sector. Some of the poor health literacy and poor health seeking behaviors evident in the population can be traced back to cultural and religious beliefs that heavily contribute to fragmentation in healthcare delivery systems. While a majority of the population are educated, cultural and religious leanings play a role in the decisions about when and where to seek medical attention. The options are: Hospitals, community pharmacies, medicine stores, traditional healing homes/herbalists, and spiritual healing centers/prayer houses. Anambra scores high on infant immunization compared to other Nigeria States however, adult immunization is low. With the increased penetration of infant immunization awareness and program implementation, there is great potential for adult immunization programs to succeed in Anambra State.

Study sites within Anambra State were identified through local constituents by the regional zones Adazi-Ani, Onitsha, and Awka. Sites were contacted directly for permission and participation in the survey.

### Sampling Method

All facilities within Anambra State were eligible to participate and only one site declined to participate due to time constraints. Because of the convenience sample method, the study relied on participants who were readily available during the data collection period, creating the potential for participation bias. This was a known limitation but deemed necessary as our sampling method with the given time and resource limitations.

### Data Analysis

Data analyses were performed using Microsoft Excel for descriptive statistics and R software for the logistic regression, with a statistical significance level of 5%. Subgroup analysis was performed for the baseline knowledge questionnaire to determine if there were any differences in correct responses based on demographics like: Institution type, profession, age, sex, religion and parental status. The study was not sufficiently powered to detect differences in the subgroup analysis.

## Results

### Demography

Responses were collected from 137 Nigerian health workers; 44% were nurses, 14% physicians, 6% pharmacists and 31% other health workers as shown in Table 1. The majority of respondents were female (69%), between 18 and 39 years of age (78%), from urban settings (82%), and identified as having Christian religious beliefs (97%). Responses were obtained from 11 sites with a balance of private (37%), public (35%) and missionary institutions (25%).

**Table 1:** Demographics.

	N (%)

**Age (years)**	
18–29	58 (42)
30–39	49 (36)
40–49	12 (9)
50–59	12 (9)
60 or greater	4 (3)
**Sex**	
Female	95 (69)
Male	42 (31)
**Home Setting**	
Urban (e.g. city)	112 (82)
Rural	25 (18)
**Religion**	
Christian	133 (97)
**Parental Status**	
Children	66 (48)
No Children	70 (51)
**Profession**	
Nurse	60 (44)
Physician	19 (14)
Pharmacist	8 (6)
Other health workers:	43 (31)
Medical lab scientist	22 (16)
Physical therapist	4 (3)
Midwife	3 (2)
Microbiologist	3 (2)
Dietician	2 (2)
Dentist	1 (1)
**Institution**	
Private	51 (37)
Public	48 (35)
Mission	34 (25)

### Perceived Barriers

**Figure 1:**
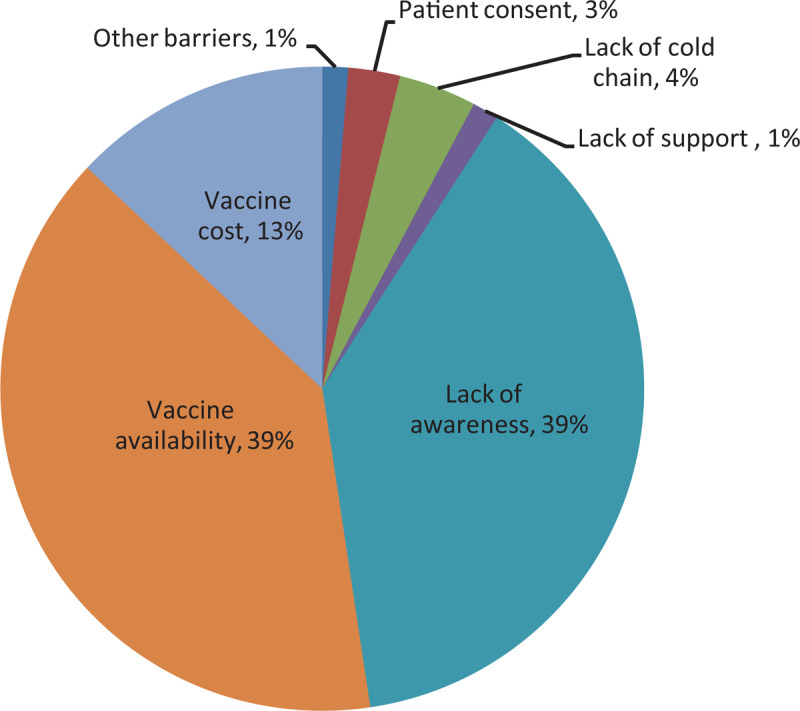
Most Significant Barrier.

The most significant barriers identified were lack of awareness (39%), vaccine availability (39%), and cost (13%) (Figure 1). The comprehensive list of other identified barriers is shown in Table 2. Broadly, the barriers mentioned can be grouped into the following categories:

- Educational – barriers regarding the public and health workers being misinformed
- Access – barriers regarding accessing the vaccine, including cost
- Practical – barriers regarding product supply storage and potency
- Other – e.g. religious beliefs, vaccine schedule compliance, etc.

**Table 2:** Barriers Identified by Category.

	Categorized Barriers

**Educational**	Provider training Insufficient knowledge Ignorance Myths, misconceptions Superstitions
**Access**	Manpower More trained personnel Distance to clinic, transportation Rural outreach
**Practical**	Storage Potency Expiration Vaccine transport Preservation
**Other**	Vaccine side effects Vaccine schedule compliance Patient acceptability Religious beliefs

* Inclusive of key words/phrases written by respondents in the free response portion of survey.

While patient acceptability of vaccine was ranked low as a significant barrier, when given the opportunity for free text comments, respondents cited some concerns about vaccine misconceptions (**Box 1**).

**Box 1: Direct Quotes (representative examples).**

- “Generally people in the rural areas do not like to receive vaccines due to superstition”
- “Adequate awareness dispels myths, misconceptions and rumors of vaccine ill effects of vaccinations”
- “Some people have the notion that it reduces fertility”
- “There is speculation that the vaccine for polio myelitis contain some substance that causes infertility/sterility in male, thereby reducing population. [Is this] True or False?”
- “Religious background could be a factor negating vaccine administration”
- “Religious beliefs should also be considered in administering the vaccine. Media awareness may also go a long way in helping”

**Figure 2:**
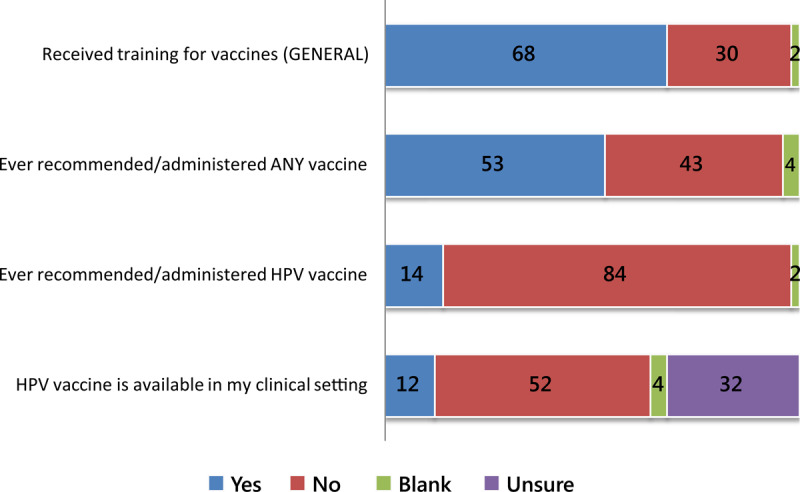
Experiences & Practices (%).

Figure 2 shows responses to select survey items regarding experiences and practices by percentage. More than half (53%) of all respondents reported having ever administered or recommended any vaccine, while only 14% had ever administered or recommended the HPV vaccine. When asked if the HPV vaccine was available in their clinical setting, the majority (52%) answered “no,” 32% were unsure and only 12% of respondents answered “yes.” Respondents were further asked if the vaccine was obtainable in Nigeria, 40% said that it was, while about half (48%) were unsure if the HPV vaccine was obtainable in Nigeria and 7% said it was not. The majority of respondents (79%) agreed that health workers should offer the HPV vaccine; specifically, 32% strongly agreed, 47% agreed, 10% were neutral, 2% disagreed, 2% strongly disagreed, 7% had no response.

### Baseline Knowledge

Baseline knowledge about appropriate use of the HPV vaccine was split, only 41% of respondents answered “yes” they knew who is eligible for the vaccine, half (49%) of the respondents admitted to not knowing (25% answered “no” and 24% answered “unsure”). See Table 3 for specific survey questions with total correct responses and responses broken down by sex. Female responses when compared to male responses were less likely to indicate that they could identify target patients for vaccination and were more likely to answer key vaccination questions incorrectly compared to male respondents. For example, when asked if HPV is sexually transmitted, females were more likely to answer incorrectly compared to males (*p = 0.008*). When asked if HPV is an infection that only affects women, females were again more likely to answer incorrectly compared to males (*p = 0.004*).

**Table 3:** Baseline Knowledge Responses Total and By Sex.

Survey question	Total responses	Correct n (%)	Not Correct		p-value **Correct vs not correct by group

			Incorrect n (%)	Not sure n (%)	

HPV puts one at risk for acquiring cervical cancer	137	111 (81)	8 (6)	10 (7)	
Males	40	38 (95)	1 (3)	1 (3)	0.069
Females	89	73 (82)	7 (8)	9 (10)	
HPV is sexually transmitted	137	81 (59)	22 (16)	28 (20)	
Males	41	32 (78)	5 (12)	4 (10)	0.008
Females	90	49 (54)	17 (19)	24 (27)	
HPV is an infection that only affects women	137	42 (30)	65 (47)	22 (16)	
Males	41	19 (46)	12 (29)	10 (24)	0.004
Females	88	23 (26)	53 (60)	12 (14)	

* Columns/rows may not add to 100% due to missing data.

Overall, statistical analysis showed that responses stratified by select characteristics (i.e. based on age, sex, years in practice, experience recommending HPV vaccine, institution type) were not significantly different, except for some correlation with the sex of respondent as mentioned above.

## Discussion

This study helps to inform a gap in available literature by providing Nigerian health workers perspective of the barriers to widespread use of the HPV vaccine. Identifying the barriers is the first step in creating a strategy that will aid in national program planning. Challenges to HPV vaccination have been previously described for sub-Saharan Africa as a region, which include inadequate infrastructure, limited health worker training, vaccine cost and cold capacity constraints [15]. Widespread use of the vaccine is achievable, as previously described in Rwanda, the first low-income country to implement a national HPV vaccination program achieving 98% vaccine coverage through government ownership, industry partnership, school-based delivery and social mobilization [15]. Our findings are consistent with previous literature while additionally providing granular perspectives adding to the limited knowledge about vaccine uptake in Nigeria.

### Awareness

The comprehensive list of perceived barriers identified in this study is consistent with previous literature, though the emphasis of awareness over cost was noteworthy. Our results align with previous studies based in Nigeria which demonstrated that the community may be poorly informed about cervical cancer disease and prevention [16,17,18,19]. Improving awareness of cervical cancer and the HPV vaccine may or may not be a promising strategy on its own. Looking at parallels from Zambia, Nyambe et al described a significant association between having awareness of cervical cancer and actions such as practicing screening and having their daughter(s) vaccinated [20]. A comprehensive approach to tackling the barriers concurrently may lead to better success.

It is unclear why female respondents were statistically significantly more likely to answer select baseline knowledge questions incorrectly, we cannot rule out that this finding occurred by chance, but the possible relationship may warrant further investigation. It is also possible that because women were more likely to work at religious institutions the difference in baseline knowledge may be a reflection of the difference in workplace distribution and not females in general.

### Availability

Vaccine availability was tied with awareness as the most significant barrier to HPV vaccination. Results regarding the lack of vaccine availability were consistent throughout the study questionnaire responses (Figure 2). As is typical in developing countries, the majority of the country’s supply of HPV vaccine may likely be available first in federal institutions located in urban areas, but our survey did not capture responses from such institutions.

Free responses in our study also identified rural outreach as a barrier, possibly contributing to the lack of availability in these areas. While it was not within the scope of this research project, an in-depth analysis may be warranted to better understand the distribution of HPV vaccine supply to reveal the complexity of the availability barrier.

### Cost

Despite the perceived cost barrier of the HPV vaccine, Umeh et al concluded that mothers in Anambra state are willing to pay [21]. This assessment compared the difference between Nigerian mothers’ willingness to pay for the HPV vaccine and estimated costs of vaccination. The assessment suggests that a government-led subsidized vaccine program may be a viable option even if associated with a copayment [21].

Anambra State Government launched the Anambra State Health Insurance Scheme in 2018. The scheme is headed by a team of locally and internationally trained healthcare professionals committed to leveraging innovative health financing solutions and existing health infrastructure to ensure health coverage. In less than 12 months, the scheme has provided primary as well as some secondary health care coverage to over 500,000 citizens. An adult immunization program designed in collaboration with manufacturers, providers and the health insurers can further reduce costs.

Collaborations with programs like GAVI may improve access to the HPV vaccine through discounts. In March 2013, for the first time, GAVI has provided support for HPV vaccine [22]. Seven countries approved for HPV demonstration program support – including sub-Saharan Africa – have piloted programs to deliver the HPV vaccine [22]. Vaccine manufacturers have negotiated lowering the cost of vaccine to US$4.60 per dose in approved countries, a 96% reduction on the price in developed countries [23]. While Nigeria is not one of the seven countries currently receiving GAVI support for HPV, the country is eligible and has collaborated with GAVI for other life-saving vaccines in the past (i.e. yellow fever vaccine) [22].

When considering healthcare economics more broadly, preventative strategies like vaccination have consistently been shown to have good return on investments. The challenge for healthcare funding in developing countries continues to be competing interests. Often, allocated funds are not adequate to support preventative efforts, as seems to be the case for cervical cancer prevention.

### Study limitations

The study limitations include a small sample size, convenience sampling method that may not be representative of the larger population, and potential for participation bias. This was a known limitation but deemed necessary as our sampling method given time and resource limitations. The sample was also geographically concentrated and predominately Christian, which may not reflect experiences of religious diversity such as that in northern Nigeria. We cannot rule out the possibility of other cultural and contextual barriers playing a role in subject understandability of the survey questionnaire, but a pilot study conducted prior to this study attempted to minimize this potential limitation. This study did not address competing health interests such as other acute conditions which could have further provided insight to health priorities, but because Nigeria has plans for implementation of a national strategy specific to cervical cancer mortality we believe this study can be valuable in a larger context.

## Conclusion

Our data informs current efforts as it documents the barriers to HPV vaccination identified directly by local health workers in this community. This study aids in bringing the important topic of preventable cancer to the forefront of global health disparities.

Barriers to HPV vaccination are numerous and complex; this study documents the most significant barriers identified within this community. Despite the barriers identified, the large majority of health workers in this study agreed that the HPV vaccine should be offered. Additional support for ongoing efforts to ensure availability and accessibility (including subsidized cost) could improve the rates of administration in Nigeria and ultimately improve death rates due to cervical cancer in this population.

Future directions include creating interventions aimed at decreasing the barriers identified in this study. Future focuses should be on education, collaborations to improve access, and continued research of evidence-based strategies for vaccine introduction and wide-spread availability. Development of educational interventions for both health workers and the general public, including strengthening vaccine science in pre- and in-service education are warranted.

## Declarations

### Ethics approval and consent to participate

This study was reviewed and classified by the University of California, San Francisco’s Committee on Human Research as exempt from full committee review, and provided exempt certification, IRB Number: 13-10617.

### Consent for publication

The authors give consent for publication; this manuscript has not been published before nor has it been submitted to another journal.

### Availability of data and material

Nguyen, Anglemyer and Brock have full access to all the data in the study and take responsibility for the integrity of the data and accuracy of the data analysis.

## Additional File

The additional file for this article can be found as follows:

10.5334/aogh.2890.s1 Supplement A. Detailed breakdown of gender by profession type.

## Author Information

The Corresponding Author has the right to grant on behalf of all authors and does grant on behalf of all authors, a worldwide license to the Publishers and its licensees in perpetuity, in all forms, formats and media (whether known now or created in the future), to i) publish, reproduce, distribute, display and store the Contribution, ii) translate the Contribution into other languages, create adaptations, reprints, include within collections and create summaries, extracts and/or, abstracts of the Contribution, iii) create any other derivative work(s) based on the Contribution, iv) to exploit all subsidiary rights in the Contribution, v) the inclusion of electronic links from the Contribution to third party material wherever it may be located; and, vi) license any third party to do any or all of the above.

All authors have completed the ICMJE uniform disclosure form at www.icmje.org/coi_disclosure.pdf and declare: all authors had financial support in the form of a research grant from UCSF Global Health Sciences Kwan Fund and RISE Global Health Initiative for the submitted work; no financial relationships with any organizations that might have an interest in the submitted work in the previous three years; no other relationships or activities that could appear to have influenced the submitted work.
