# Supplementary material for: Identifying Perceived Barriers to Human Papillomavirus Vaccination as a Preventative Strategy for Cervical Cancer in Nigeria
Source: Ann Glob Health. 2020 Sep 17;86(1):118. doi: 10.5334/aogh.2890 (PMC7500222; doi:10.5334/aogh.2890)
Supplement: Supplement A. — Detailed breakdown of gender by profession type. [file agh-86-1-2890-s1.pdf]

Supplement A. Detailed breakdown of gender by profession type.

	**N=137 (%)**	**Female, 95 (69)**	**Male, 42 (31)**
Nurse	60 (44)	59	1
Medical lab scientist	22 (16)	11	11
Physician	19 (14)	1	18
Pharmacist	8 (6)	4	4
Pharmacy dispenser	4 (3)	4	0
Physical therapist	4 (3)	0	4
Midwife	3 (2)	3	0
Microbiologist	3 (2)	3	0
Dietician	2 (1.5)	2	0
Pharmacologist	1 (0.7)	0	1
Scientist	1 (0.7)	1	0
Dentist	1 (0.7)	0	1

*Missing data or “other” for 9 responses (6.5%).
